# Supplementary material for: Hybrid protein–ligand binding residue prediction with protein language models: does the structure matter?
Source: Bioinformatics. 2025 Jul 31;41(8):btaf431. doi: 10.1093/bioinformatics/btaf431 (PMC12377911; doi:10.1093/bioinformatics/btaf431)
Supplement: btaf431_Supplementary_Data [file btaf431_supplementary_data.zip › PLBS_PLM_GNN___OUP_template - supplementary materials.pdf]

Supplementary material

	Number of convolutional layers			
Ligand	1	2	4	6
ADP	**0.664 ± 0.028**	0.646 ± 0.031	0.639 ± 0.034	0.631 ± 0.039
AMP	**0.453 ± 0.064**	0.447 ± 0.062	0.443 ± 0.063	0.429 ± 0.047
ATP	**0.572 ± 0.015**	0.566 ± 0.012	0.559 ± 0.012	0.551 ± 0.015
CA	**0.485 ± 0.015**	0.458 ± 0.015	0.429 ± 0.016	0.409 ± 0.010
DNA	0.499 ± 0.035	**0.504 ± 0.027**	0.506 ± 0.028	0.510 ± 0.025
FE	0.704 ± 0.059	**0.711 ± 0.062**	0.699 ± 0.062	0.702 ± 0.062
GDP	0.667 ± 0.054	**0.686 ± 0.062**	0.645 ± 0.082	0.596 ± 0.095
GTP	0.516 ± 0.084	**0.573 ± 0.056**	0.541 ± 0.050	0.531 ± 0.054
HEME	0.634 ± 0.031	**0.636 ± 0.039**	0.632 ± 0.035	0.622 ± 0.037
MG	**0.458 ± 0.017**	0.452 ± 0.018	0.413 ± 0.020	0.384 ± 0.024
MN	**0.609 ± 0.047**	0.602 ± 0.041	0.586 ± 0.047	0.571 ± 0.049
ZN	**0.685 ± 0.016**	0.684 ± 0.015	0.661 ± 0.011	0.636 ± 0.009

Table 1. Effect of the number of graph convolutional layers with ProtT5 embeddings and cutoff distance of 6 Å. Values represent means and standard deviations of validation MCC scores from 5-fold CV for the Yu benchmark training set.

		GCN			GAT		
Ligand	Cutoff	Precision	Recall	MCC	Precision	Recall	MCC
ADP	4	0.663	0.51	0.569	0.674	0.504	0.571
	6	0.658	0.504	0.564	0.704	0.493	0.578
	8	0.656	0.536	0.581	0.674	0.55	0.597
	10	0.661	0.491	0.557	0.641	0.551	0.582
	Ensemble	0.726	0.487	0.584	0.713	0.496	0.583
AMP	4	0.49	0.449	0.45	0.398	0.561	0.449
	6	0.443	0.423	0.412	0.465	0.503	0.463
	8	0.448	0.441	0.424	0.67	0.378	0.489
	10	0.458	0.421	0.419	0.618	0.388	0.475
	Ensemble	0.54	0.395	0.445	0.632	0.39	0.482
ATP	4	0.578	0.547	0.546	0.567	0.598	0.566
	6	0.543	0.567	0.537	0.651	0.533	0.575
	8	0.554	0.558	0.538	0.62	0.556	0.572
	10	0.579	0.567	0.557	0.66	0.547	0.587
	Ensemble	0.651	0.522	0.569	0.677	0.526	0.583
CA	4	0.526	0.308	0.396	0.473	0.322	0.383
	6	0.531	0.284	0.382	0.522	0.329	0.408
	8	0.551	0.303	0.403	0.533	0.322	0.408
	10	0.595	0.304	0.42	0.524	0.332	0.411
	Ensemble	0.647	0.28	0.421	0.613	0.303	0.426
DNA	4	0.48	0.53	0.473	0.442	0.552	0.46
	6	0.476	0.541	0.476	0.459	0.579	0.483
	8	0.411	0.625	0.47	0.438	0.674	0.51
	10	0.43	0.568	0.459	0.474	0.569	0.488
	Ensemble	0.519	0.518	0.49	0.5	0.559	0.499
FE	4	0.463	0.842	0.618	0.602	0.833	0.704
	6	0.488	0.867	0.645	0.572	0.792	0.668
	8	0.467	0.825	0.614	0.594	0.817	0.692
	10	0.493	0.858	0.645	0.609	0.858	0.719
	Ensemble	0.513	0.825	0.645	0.613	0.817	0.703
GDP	4	0.756	0.608	0.665	0.851	0.588	0.696
	6	0.782	0.593	0.668	0.801	0.624	0.695
	8	0.879	0.634	0.737	0.937	0.608	0.746
	10	0.796	0.624	0.693	0.735	0.701	0.705
	Ensemble	0.896	0.577	0.71	0.922	0.613	0.744
GTP	4	0.469	0.674	0.537	0.761	0.607	0.666
	6	0.47	0.618	0.514	0.753	0.618	0.669
	8	0.553	0.64	0.575	0.731	0.64	0.67
	10	0.544	0.629	0.564	0.508	0.697	0.573
	Ensemble	0.58	0.573	0.556	0.809	0.618	0.695
HEME	4	0.716	0.7	0.689	0.715	0.676	0.675
	6	0.718	0.667	0.672	0.773	0.621	0.674
	8	0.755	0.75	0.736	0.799	0.719	0.743
	10	0.716	0.674	0.675	0.724	0.679	0.682
	Ensemble	0.775	0.648	0.691	0.79	0.624	0.685
MG	4	0.459	0.264	0.343	0.44	0.249	0.325
	6	0.443	0.276	0.344	0.471	0.264	0.347
	8	0.438	0.292	0.351	0.473	0.289	0.364
	10	0.481	0.281	0.362	0.463	0.272	0.349
	Ensemble	0.526	0.261	0.365	0.537	0.254	0.364
MN	4	0.6	0.646	0.617	0.625	0.591	0.602
	6	0.565	0.662	0.606	0.684	0.612	0.642
	8	0.596	0.603	0.594	0.608	0.616	0.607
	10	0.55	0.646	0.59	0.652	0.641	0.642
	Ensemble	0.64	0.637	0.634	0.679	0.608	0.638
ZN	4	0.64	0.691	0.66	0.667	0.683	0.67
	6	0.711	0.661	0.681	0.681	0.672	0.672
	8	0.685	0.671	0.673	0.712	0.668	0.685
	10	0.729	0.667	0.693	0.713	0.677	0.69
	Ensemble	0.755	0.655	0.699	0.746	0.663	0.699

Table 2. Comparison of GAT and GCN for ProtT5 embeddings and for different cutoff distances. Values represent scores for the Yu benchmark test set.

		AAIndex			ProtBERT			SeqVec			ESM-2		
Ligand	Cutoff	Precision	Recall	MCC	Precision	Recall	MCC	Precision	Recall	MCC	Precision	Recall	MCC
ADP	4	0.055	0.557	0.089	0.755	0.324	0.484	0.822	0.741	0.557	0.801	0.814	0.615
	6	0.075	0.436	0.115	0.728	0.340	0.487	0.827	0.752	0.575	0.802	0.788	0.590
	8	0.077	0.583	0.142	0.659	0.405	0.504	0.836	0.743	0.571	0.807	0.809	0.616
	10	0.084	0.573	0.154	0.705	0.359	0.492	0.844	0.760	0.598	0.831	0.791	0.621
	Ensemble	0.127	0.430	0.188	0.807	0.324	0.502	0.854	0.738	0.580	0.826	0.789	0.614
AMP	4	0.050	0.648	0.067	0.233	0.429	0.281	0.806	0.629	0.397	0.764	0.747	0.511
	6	0.060	0.571	0.092	0.365	0.235	0.272	0.822	0.610	0.376	0.716	0.766	0.480
	8	0.060	0.643	0.101	0.321	0.388	0.326	0.769	0.624	0.365	0.782	0.716	0.493
	10	0.058	0.737	0.108	0.313	0.370	0.313	0.824	0.628	0.407	0.808	0.702	0.499
	Ensemble	0.068	0.569	0.111	0.359	0.278	0.293	0.858	0.613	0.403	0.810	0.718	0.521
ATP	4	0.054	0.567	0.071	0.541	0.366	0.428	0.800	0.717	0.511	0.781	0.800	0.581
	6	0.067	0.573	0.106	0.441	0.490	0.443	0.782	0.727	0.506	0.786	0.792	0.577
	8	0.073	0.470	0.106	0.449	0.484	0.445	0.783	0.732	0.512	0.821	0.778	0.597
	10	0.075	0.516	0.116	0.445	0.539	0.468	0.806	0.721	0.520	0.805	0.797	0.601
	Ensemble	0.092	0.454	0.136	0.552	0.440	0.476	0.825	0.712	0.525	0.822	0.783	0.604
CA	4	0.038	0.615	0.110	0.481	0.248	0.339	0.691	0.621	0.303	0.710	0.661	0.367
	6	0.038	0.641	0.112	0.543	0.233	0.350	0.741	0.605	0.317	0.750	0.660	0.400
	8	0.045	0.575	0.122	0.598	0.246	0.378	0.713	0.613	0.310	0.744	0.665	0.401
	10	0.048	0.611	0.134	0.547	0.237	0.354	0.730	0.616	0.327	0.730	0.669	0.394
	Ensemble	0.054	0.558	0.139	0.661	0.214	0.372	0.776	0.601	0.334	0.777	0.647	0.404
DNA	4	0.094	0.693	0.138	0.354	0.461	0.363	0.627	0.683	0.305	0.681	0.792	0.460
	6	0.103	0.629	0.145	0.352	0.503	0.379	0.652	0.668	0.320	0.692	0.771	0.456
	8	0.118	0.597	0.168	0.323	0.562	0.381	0.656	0.667	0.322	0.677	0.808	0.467
	10	0.124	0.622	0.184	0.355	0.499	0.380	0.656	0.677	0.332	0.673	0.829	0.477
	Ensemble	0.124	0.584	0.175	0.405	0.455	0.393	0.676	0.659	0.334	0.705	0.788	0.486
FE	4	0.061	0.808	0.194	0.557	0.692	0.615	0.772	0.805	0.576	0.759	0.916	0.657
	6	0.089	0.675	0.221	0.535	0.767	0.635	0.758	0.809	0.564	0.727	0.931	0.626
	8	0.090	0.775	0.241	0.535	0.767	0.635	0.765	0.825	0.587	0.757	0.903	0.643
	10	0.084	0.808	0.237	0.458	0.825	0.609	0.761	0.838	0.593	0.769	0.928	0.678
	Ensemble	0.121	0.733	0.279	0.544	0.725	0.622	0.787	0.801	0.588	0.765	0.916	0.664
GDP	4	0.089	0.531	0.130	0.624	0.546	0.566	0.919	0.740	0.635	0.934	0.828	0.754
	6	0.115	0.474	0.163	0.677	0.552	0.595	0.954	0.741	0.662	0.919	0.814	0.726
	8	0.156	0.479	0.215	0.675	0.526	0.580	0.905	0.753	0.640	0.936	0.823	0.750
	10	0.145	0.593	0.230	0.603	0.557	0.561	0.930	0.751	0.657	0.935	0.836	0.764
	Ensemble	0.201	0.438	0.249	0.768	0.495	0.603	0.948	0.736	0.651	0.951	0.816	0.754
GTP	4	0.088	0.551	0.129	0.694	0.483	0.563	0.899	0.734	0.611	0.876	0.843	0.718
	6	0.122	0.517	0.180	0.658	0.562	0.591	0.926	0.768	0.675	0.864	0.837	0.701
	8	0.133	0.506	0.192	0.643	0.506	0.552	0.876	0.749	0.613	0.841	0.830	0.671
	10	0.097	0.562	0.149	0.613	0.551	0.562	0.934	0.768	0.682	0.876	0.843	0.718
	Ensemble	0.184	0.449	0.235	0.789	0.506	0.618	0.938	0.751	0.664	0.889	0.838	0.725
HEME	4	0.105	0.612	0.131	0.577	0.472	0.493	0.830	0.751	0.575	0.868	0.878	0.746
	6	0.117	0.617	0.156	0.593	0.495	0.514	0.829	0.742	0.565	0.869	0.875	0.744
	8	0.142	0.681	0.214	0.617	0.566	0.564	0.862	0.750	0.602	0.881	0.874	0.755
	10	0.136	0.616	0.190	0.552	0.547	0.519	0.849	0.764	0.607	0.876	0.885	0.761
	Ensemble	0.153	0.572	0.206	0.699	0.476	0.554	0.878	0.743	0.606	0.891	0.871	0.762
MG	4	0.024	0.546	0.068	0.447	0.244	0.325	0.672	0.602	0.264	0.720	0.636	0.346
	6	0.024	0.482	0.065	0.480	0.227	0.325	0.704	0.601	0.287	0.716	0.638	0.345
	8	0.032	0.437	0.083	0.412	0.264	0.324	0.700	0.611	0.298	0.713	0.643	0.350
	10	0.029	0.482	0.078	0.477	0.232	0.328	0.700	0.606	0.291	0.725	0.641	0.357
	Ensemble	0.040	0.417	0.098	0.519	0.219	0.333	0.740	0.597	0.306	0.741	0.634	0.359
MN	4	0.051	0.776	0.165	0.511	0.506	0.502	0.786	0.715	0.496	0.776	0.813	0.588
	6	0.076	0.624	0.191	0.552	0.473	0.505	0.764	0.715	0.477	0.789	0.790	0.580
	8	0.079	0.667	0.204	0.544	0.498	0.514	0.751	0.729	0.480	0.827	0.772	0.597
	10	0.085	0.646	0.210	0.543	0.527	0.529	0.797	0.715	0.506	0.815	0.785	0.599
	Ensemble	0.106	0.662	0.243	0.591	0.481	0.527	0.805	0.709	0.505	0.819	0.781	0.599
ZN	4	0.096	0.895	0.268	0.640	0.578	0.602	0.789	0.785	0.574	0.855	0.825	0.679
	6	0.131	0.798	0.303	0.648	0.579	0.607	0.809	0.779	0.587	0.854	0.825	0.679
	8	0.152	0.810	0.331	0.640	0.609	0.618	0.809	0.790	0.598	0.858	0.825	0.683
	10	0.146	0.852	0.334	0.636	0.612	0.618	0.803	0.801	0.604	0.849	0.830	0.678
	Ensemble	0.165	0.816	0.349	0.687	0.570	0.621	0.842	0.781	0.620	0.878	0.816	0.691

Note : Here, embeddings are compared for the GAT model.

Table 3. Comparison of different embeddings with different cutoff distances. Values represent scores for the Yu benchmark test set.

Model architecture	Hyperparameters	Validation MCC
MLP	3 layers, 64 units, dropout 0.5	0.560 ± 0.037
	3 layers, 256 units, dropout 0.5	0.609 ± 0.032
	3 layers, 512 units, dropout 0.5	0.618 ± 0.033
	1 layer, 64 units, dropout 0.1	0.602 ± 0.034
	1 layer, 256 units, dropout 0.1	0.629 ± 0.035
	1 layer, 512 units, dropout 0.1	**0.639 ± 0.036**
Linear SVM	C = 1.0	0.400 ± 0.023
	C = 10.0	0.461 ± 0.042
	C = 0.1	0.387 ± 0.018
Random Forest	`n_estimators = 100 , max_features = all_features`	0.310 ± 0.035
	`n_estimators = 100 , max_features = 0.5`	0.307 ± 0.029

Table 4. Models and hyperparameters used to select the sequence baseline. Values represent the means and standard deviations of validation MCC scores from 5-fold CV for the ADP ligand training dataset.

**Embedding**	**AAIndex**	**SeqVec**	**ProtBERT**	**ProtT5**	**ESM-2**
**Dataset**	-	UniRef50	BFD	BFD, UniRef50	UniRef50
**Number of Parameters**	-	93M	420M	3B	3B
**Embedding dimension**	566	1024	1024	1024	2560

Table 5. Comparison of embeddings.

**Hyperparameter**	**Values**
Number of units in GNN layers	64, 256, 512, 1024
Learning rate	3e-4, 1e-3
Weight decay	1e-5, 1e-2
Dropout rate	0, 0.3, 0.5
Number of attention heads in GAT	1, 2, 4
Residual connections	True, False
Batch normalization	True, False

Table 6. Hyperparameter values tried in manual tuning.

Model architecture	Dropout rate	Validation MCC
GCN	0	0.679
	0.1	0.669
	0.3	0.676
	0.5	**0.687**
GAT	0	0.594
	0.1	0.678
	0.3	0.698
	0.5	**0.705**

Table 7. Different dropout rates tried during hyperparameter tuning for GCN and GAT models. The dataset used is the ADP training dataset from the Yu benchmark, where a random validation split was used with the same random seed. The models use a threshold of 8 Å for graph construction, and use ProtT5 embeddings for node features. Values represent the highest validation MCC score obtained after training on 2000 epochs (early stopping). The table indicate that a dropout rate of 0.5 yields the best performance for both architectures. This finding aligns with existing research, where high dropout rates have been shown to enhance the generalization in GNNs [Luo et al., Cai et al., 2020, Wang et al., 2023a].

Dataset	Sequences	Binding residues	Non-Binding residues	Missing protein graphs
DNA_Train_573	573	14479	145404	18
DNA_Test_129	129	2240	35275	2
DNA_Test_181	181	3208	72050	18
RNA_Train_495	495	14609	122290	36
RNA_Test_117	117	2031	35314	10

Table 8. Protein-DNA/RNA benchmarks summary. From GraphBind we used the protein-DNA benchmarking set consisting of a training set DNA_Train_573 and a test set DNA_Test_129 , and we employed the protein-RNA benchmarking set consisting of a training set RNA_Train_495 and a test set RNA_Test_117. From GraphSite, we used the protein-DNA benchmarking test set DNA_Test_181, and we trained the model on the same protein-DNA set DNA_Train_573 from GraphBind. All protein-DNA/RNA benchmarks were downloaded in FASTA format, and underwent the same preprocessing strategy used for the Yu benchmark. We thus had to discard protein sequences with a high mismatch between the sequence from the benchmark and the sequence of residues from PDB.

Dataset	Method	AUC	AUPR	MCC
DNA_Test_129	GraphBind	0.928	0.519	0.499
	GraphSite	0.919	0.502	-
	EquiPNAS	**0.943**	**0.582**	-
	GeoBind	0.940	-	**0.526**
	GAT8 + ProtT5 (ours)	0.922	0.510	0.488
DNA_Test_181	GraphBind	0.904	0.339	0.392
	GraphSite	0.903	0.336	**0.397**
	EquiPNAS	**0.921**	**0.393**	-
	GAT8 + ProtT5 (ours)	0.898	0.337	0.364
RNA_Test_117	GraphBind	0.854	-	0.322
	GeoBind	0.874	-	**0.373**
	EquiPNAS	**0.887**	**0.320**	
	GAT8 + ProtT5 (ours)	0.810	0.261	0.292

Table 9. Comparison with existing methods - protein-DNA/RNA benchmarks. Values represent the scores for the test sets from the benchmark.

Sequences	Binding residues	Non-binding residues	Percentage of binding residues
2516	122784	618043	16.57%

Table 10. Summary of Curated dataset from PDBBind.

Embedding	Model	Best validation MCC
**Seqvec**	Sequence	0.478 ± 0.051
	GAT8	0.531 ± 0.054
	Δ_{abs}	0.053 ± 0.009
	Δ_{rel}	0.112 ± 0.020
	P-Value	0.0001
**ESM-2**	Sequence	0.623 ± 0.037
	GAT8	0.625 ± 0.042
	Δ_{abs}	0.002 ± 0.009
	Δ_{rel}	0.003 ± 0.014
	P-Value	0.3203

Table 11. Performance of Sequence Baseline and GAT8 models with SeqVec and ESM-2 embeddings on the new dataset constructed from PDBBind. Values represent the highest validation MCC scores obtained during training of the five different splits. The P-values are obtained from the statistical t-tests performed on the Δ_{rel} values.

	Training sets		Independent test sets		
Ligand	Sequences	% of Binding Residues	Sequences	% of Binding Residues	Performance
ATP	221	4.01%	50	3.74%	Medium
ADP	296	3.74%	47	3.26%	High
AMP	145	3.48%	33	3.65%	Medium
GDP	82	4.03%	14	4.44%	High
GTP	54	3.39%	7	4.55%	High
CA	965	1.68%	165	1.44%	Low
ZN	1168	1.47%	176	1.53%	High
MG	1138	1.09%	217	1.17%	Low
MN	335	1.31%	58	1.34%	High
FE	173	1.60%	26	1.30%	High
DNA	335	8.31%	52	5.66%	Medium
HEME	206	8.09%	27	6.30%	High

Table 12. Yu benchmark - Effect of dataset characteristics on performance. The table summarizes fundamental dataset statistics, including the number of sequences and the proportion of binding residues relative to the total residue count. It also compares these characteristics with the performance of the T5 GAT8 model (as shown in Table 3) across various ligand types. Performance levels are categorized as High ($MCC \geq 0.5$), Medium ($0.45 \leq MCC < 0.5$), and Low ($MCC < 0.45$). Based on the data, there is no evident relationship between these basic characteristics and model performance. This indicates that simple dataset metrics alone do not sufficiently explain performance variability. Other factors, such as the degree of similarity between training and test set proteins or binding sites, may have a greater impact. A more comprehensive analysis, potentially requiring a larger dataset, is therefore needed to explore these influences further.
